# Prevalence of dual sensory impairment in Australia: a nationally representative population-based survey of Indigenous and non-Indigenous Australians

**DOI:** 10.1016/j.lanwpc.2026.101897

**Published:** 2026-06-05

**Authors:** Bamini Gopinath, Oonagh Macken, Richard Kha, Gary Low, Yasemin Kapucu, John Newall, Colina Waddell, Tim Fricke, Eleanor Yang, Mayuri Indrakumar, Diana Tang, Angus Turner, Lisa Keay, Gerald Liew, Paul Mitchell

**Affiliations:** a Macquarie University Hearing, Macquarie University, Sydney, NSW, Australia; b Centre for Vision Research, Westmead Institute for Medical Research, University of Sydney, Sydney, NSW, Australia; c Brien Holden Foundation, Sydney, NSW, Australia; d School of Optometry and Vision Science, UNSW, Sydney, NSW, Australia; e Australian College of Optometry, Melbourne, VIC, Australia; f Lions Outback Vision, Lions Eye Institute, Nedlands, WA, Australia; g Centre for Ophthalmology and Visual Science, The University of Western Australia, Perth, WA, Australia

**Keywords:** Dual sensory impairment, Hearing impairment, Vision impairment, Population-based survey, Prevalence, Risk factors, Indigenous

## Abstract

**Background:**

The prevalence of co-occurring vision and hearing impairment (Dual Sensory Impairment, DSI) is expected to increase with the global ageing population. This national survey aimed to assess the prevalence and risk factors for objectively measured DSI in adults.

**Methods:**

Stratified, multi-stage random cluster sampling of 30 sites was conducted. DSI cases included persons with bilateral vision impairment (presenting distance visual acuity <6/12 in the better eye) and either, any (>25 dB hearing level, dB HL, in the unaided better ear) or moderate to worse bilateral hearing impairment (>40 dB HL, unaided better ear).

**Findings:**

4519 participants aged 50+ years underwent the eye examination and 3573 (79.1%) also completed the ear examination. Age-standardized prevalence of DSI was 2.5% for any hearing impairment, and 1.3% for moderate or worse hearing impairment. DSI prevalence for the >25 dB HL and >40 dB HL categories, increased by ∼13- and 9-fold (p < 0.0001), respectively, from 50 to 80+ years. Age and not having private health insurance were associated with greater likelihood of DSI (for any hearing impairment) in Indigenous and non-Indigenous participants. Not having private insurance accounted for 34.0% and 86.2% of the total DSI burden in non-Indigenous and Indigenous adults, respectively. In non-Indigenous adults, living in remote/very remote areas was a risk factor: OR 2.87 (95% CI 1.27–5.96).

**Interpretation:**

DSI prevalence rises sharply with age in Australian adults. Socioeconomic disadvantage and geographic inequities account for a substantial proportion of Australia’s DSI burden.

**Funding:**

Australian Commonwealth Government Department of Health; Martin Lee Centre for Innovations in Hearing Health.

**Research in context**

**Evidence before this study**

We surveyed PubMed and government reports in 2020 and identified that only one population-based study has examined the prevalence of objectively measured dual sensory impairment (DSI), that is, coexisting vision impairment (VI) and hearing impairment (HI) in older Australians to date, and none provided data on DSI in Indigenous adults. Contemporary data on objectively measured DSI are needed, particularly for Indigenous Australian adults, where there is a known three-fold higher rate of VI, and suspected but not precisely known, higher rates of HI. The Australian Eye and Ear Health Survey (AEEHS) was conducted to address knowledge gaps in sensory loss.

**Added value of this study**

The AEEHS found that among Australian adults aged 50+ years, the age-standardized prevalence of DSI was 2.5% for any bilateral VI (presenting distance visual acuity <6/12 in the better eye) together with any unaided bilateral HI (>25 dB hearing level, dB HL, in the better ear), and 1.3% for moderate or worse unaided HI (>40 dB HL). Crude DSI prevalence rates rose steeply with age, approximately 13-fold and 9-fold between ages 50 and 80+ years, for DSI defined by >25 dB HL and >40 dB HL, respectively. These data highlight the strong influence of aging on sensory decline. Men had higher DSI prevalence than women for the >25 dB HL category, though sex differences diminished with more severe hearing loss.

Indigenous participants had a greater prevalence of DSI than non-Indigenous participants across both hearing loss categories. The elevated DSI prevalence rates observed among Indigenous adults under 70 years, indicates earlier onset and disproportionate sensory impairment burden. Multivariable analyses revealed that older age, residence in remote/very remote areas, and lack of private health insurance independently predicted higher odds of DSI (for >25 dB HL category) in non-Indigenous adults: OR 1.16 (95% CI 1.13–1.2); OR 2.87 (95% CI 1.27–5.96); and OR 2.27 (95% CI 1.44–3.6), respectively. In Indigenous adults, older age and lack of private health insurance were significantly associated with prevalent DSI: OR 1.06 (1.01–1.11) and OR 9.39 (95% CI 1.84–172.2), respectively. The population attributable risks indicate that lack of private insurance accounts for 34.0% and 86.2% of the total DSI burden (for >25 dB HL) in non-Indigenous and Indigenous adults, respectively.

**Implications of all the available evidence**

This study provides the most contemporary, nationally representative estimates of prevalence of objectively measured DSI in Australian adults, and the first epidemiological data for Indigenous Australians. Findings reveal that DSI prevalence rises sharply with age, highlighting the strong influence of aging on sensory functioning and underscoring the need for integrated hearing and vision screening within primary care and chronic disease programs. The substantially higher DSI prevalence among Indigenous adults, particularly those under 70 years, points to earlier onset and a disproportionate sensory impairment burden, underscoring the importance of culturally safe and accessible sensory health services for this vulnerable community.

Geographic disparities and socioeconomic disadvantage, particularly higher odds of DSI among individuals living in remote areas and those without private insurance, reflect systemic inequities in access to preventive and rehabilitative care. These findings emphasize the need to better strengthen community-based and outreach models, including telehealth, to improve service reach and equity of access. The population-attributable risk estimates demonstrate that lack of private insurance and limited access to affordable services account for a considerable proportion of the national DSI burden. Addressing these economic and structural determinants through targeted policy and service innovations is essential to reduce inequities and promote sensory health across all populations in Australia.

## Introduction

Impaired vision and hearing are common conditions among adults and can occur separately or concurrently. Vision impairment (VI) results in reduced independence, increases use of care services, reduces quality of life, and increases risk of depression and mortality.^1^ Hearing impairment (HI) leads to poorer quality of life, mental health, and relationships, and increases reliance on community and informal supports.^2^ Concurrent VI and HI is termed deafblindness or dual sensory impairment (DSI). The impact of DSI on a person’s life is more severe than that of having only vision or hearing loss. That is, adults who have DSI face a number of unique challenges not experienced by others with different types of disability, as they may not be able to compensate for the loss of one sense with the other.^1^^,^^3^^,^^4^

A worldwide exponential rise in DSI has been attributed to ageing demographics with increasing prevalence of age-related sensorineural hearing loss, cataract, glaucoma, and macular degeneration among older adults.^5^^,^^6^ However, estimates of the prevalence of DSI in community residing adults has ranged widely and these extremes often reflect use of different samples and age ranges, different measures of sensory function (objective or self-report), and different definitions of impairment. Typically, the reported number of individuals with DSI is underestimated.^1^^,^^3^ Moreover, DSI prevalence in Indigenous communities is not well documented, as it can be challenging to collect health data from Indigenous populations who live in geographically remote areas, and further there may be cultural and other social barriers to participation in research.^7^^,^^8^ Extrapolating disease rates from largely non-Indigenous populations can underestimate the prevalence of disease burden in marginalized communities, resulting in inadequate understanding of, and insufficient interventions to help the most vulnerable groups.^7^^,^^8^

The Australian Eye and Ear Health Survey (AEEHS) was conducted to address these critical knowledge gaps and is the first national survey to focus on evaluating both eye and ear health simultaneously in a representative sample of Australians. Specific research questions were: (1) What is the crude and age-standardised prevalence of objectively measured DSI among Australian adults aged 50+ years, overall and stratified by age group and Indigeneity (descriptive statistics)? (2) Which demographic, structural and medical factors independently predict higher odds of DSI in Australian adults aged 50+ years, and how strongly do they predict DSI after multivariable adjustment (predictive statistics)? These epidemiological data are important for enabling active case-finding of adults with DSI and to link these individuals to appropriate services and supports. Further, these data are critical to answer the call by the Lancet Global Health Commission on Global Eye Health to enhance the focus on DSI to support collaboration in research, clinical care, and social inclusion.

## Methods

### Study design and participant recruitment

The AEEHS was designed as the first nationally representative study to provide comprehensive data on vision and hearing loss in Australia. Input from Indigenous co-investigators and team members were incorporated into the AEEHS design and conduct from study conception, including Indigenous governance and advisory input to ensure culturally safe approaches across design, recruitment and conduct. This included consultation with Indigenous Elders and organisations, including Aboriginal Community Controlled Health Organisations (ACCHOs), to guide community engagement, recruitment and data collection. The AEEHS enrolled Indigenous and non-Indigenous Australians aged 50 years or older, from August 2022 to March 2025. Stratified multi-stage random cluster sampling was used to select 30 geographical target sites in Australia to provide a representative sample of the nation as a whole, with sites from every state and territory.

Details of survey methodology are provided elsewhere.^9^ In short, this sampling strategy utilised data from the 2021 Australian Census, which divides all regions of Australia into Statistical Areas (SA) based on population and size of the geographical area. Statistical Area Level 2 (SA2) was used as the unit for random selection, defined as a medium-sized general-purpose area that represents a community that interacts together socially and economically.^9^ Stratification by geographic location (i.e., state and territory) was first conducted to ensure that the sample of 30 SA2 sites would be representative of the geographic distribution in which most Indigenous and non-Indigenous Australians lived. A second level of stratification was employed based on Indigenous status to ensure adequate sampling of Indigenous participants. This was necessary as fully random sampling would not result in adequate representation of Indigenous participants, who comprise around 2% of the Australian population aged 50+ years.^10^

Individual, in-person, doorknocking was the primary method of recruitment, with adjustments as required to maintain cultural appropriateness within local Indigenous communities. In communities where door-to-door recruitment was considered culturally inappropriate after consultation with Indigenous elders and health organisations, alternative methods included recruitment from concurrent Indigenous health clinics, word-of-mouth, or telephone recruitment from community lists.

### Participant examination

Participant examinations were conducted in testing venues located at each site. These included medical clinics, Indigenous health organisations, community centres and vacant commercial properties. The testing protocol consisted of a comprehensive vision and hearing assessment, including eye and ear imaging. Testing was conducted over four stations. An interviewer-administered questionnaire was used to collect sociodemographic, medication, medical/surgical history, ocular and otological history data. Sex was defined as a biological variable (male or female) based on participant self-report. Indigenous status was defined based on participant self-identification as Aboriginal and/or Torres Strait Islander, or non-Indigenous. Diabetes was defined from blood sugar, history or use of diabetic medications.

### Assessment of sensory loss

Standardised eye examinations were conducted by trained eye health professionals. The eye examination consisted of visual acuity (VA) testing, autorefraction, ocular biometry, lensometry, tonometry, visual field examination, anterior segment examination, mydriatic ultrawide-field retinal photography, fundus auto-fluorescence, optical coherence tomography (OCT) and OCT angiography. Presenting distance VA was measured for each eye using an electronic ETDRS logMAR chart at 4 m testing distance in well-lit room conditions. If presenting VA was <6/9.5 in one or both eyes, pinhole testing, auto-refraction and subjective refraction were performed to obtain best-corrected VA. Any bilateral VI was defined as presenting distance VA of <6/12 in the better eye and included all individuals with blindness.^11^

Unaided pure-tone audiometry was performed using the Avant A2D audiometer with passive noise-reducing headphones (RadioEar DD65v2). A Hughson-Westlake staircase procedure, in conjunction with 40 dB of contralateral masking, was implemented to establish thresholds in each ear across six frequencies. Noise levels were assessed by a Bruel and Kjaer type 2250 sound level meter throughout the examination. HI was determined as the four-frequency pure-tone average (PTA) of audiometric hearing thresholds at 500, 1000, 2000 and 4000 Hz. HI was stratified as mild (>25–40, decibels hearing level, dB HL), moderate (>40–60 dB HL), severe (>60–80 dB HL), and profound (>80 dB HL). Severity of HI in the better ear was used to classify the severity of bilateral HI. Any bilateral HI was defined as hearing thresholds >25 dB HL. Cases of DSI included persons with any bilateral VI, together with either any bilateral HI (>25 dB HL) or moderate or worse bilateral HI (>40 dB HL).

### Statistical analysis

Mean and standard deviation were used to describe normally distributed continuous variables. Counts and percentages were used to describe categorical variables. Crude prevalence was calculated by the number of cases of DSI divided by the total surveyed population within each stratum. The rates of each stratum were age-standardised against the Australian population stratified by Indigenous status. Specifically, age-specific prevalence within each stratum (Indigenous and non-Indigenous) were calculated and then age weights from the relevant Australian reference population were applied to produce a single summary prevalence estimate for adults aged 50+. These age-standardized estimates are read as the expected prevalence in the reference 50+ population if the study’s age-specific rates applied, rather than the raw observed prevalence in the study sample. Age-standardising to the Australian population stratified by Indigenous status means the study’s prevalence estimates were adjusted so Indigenous and non-Indigenous prevalence rates are not driven by differences in age structure between the sample groups i.e., age standardization minimises the confounding effects of age.^12^ Because DSI increases sharply with age, this adjustment supports fairer comparisons than crude prevalence, which can be misleading if one group is older on average.

We also adjusted for the sampling probability by standardising remoteness in addition to age, applying Australian Institute of Health and Welfare principles for measuring the gap between Indigenous and non-Indigenous Australians.^12^ Multivariable logistic regression models were employed with outcomes: 1) any bilateral VI and any bilateral HI with definition of >25 dBHL; and 2) bilateral VI and moderate or worse bilateral HI with definition of >40 dBHL. Akaike Information Criterion and Bayesian Information Criterion were used to determine the best fit model, as well as Variance Inflation Factor to check for multicollinearity and residual plots and tests for normality and outliers.

The population-attributable risk (PAR) was calculated for statistically significant risk factors, using the following equation: PAR = p × (OR − 1)/[p × (OR − 1) + 1], where p is prevalence of the factor, and OR was derived from the multivariable logistic regression models.

The “Table 2 fallacy” is the risk of treating all coefficients from a single multivariable regression table as if they represent causal (total) effects, even though some variables may be confounders, mediators, or lie on different causal pathways and hence, cannot all be interpreted causally within one model.^13^ To reduce this risk, Directed Acyclic Graphs (DAGs) and causal mediation analysis was used to map hypothesised pathways, identify mediators (indirect effects), and determine when separate models were needed to estimate total effects correctly. In this approach, age, diabetes, and private health insurance were treated as primary exposures for DSI. DAGs were used to specify likely causal relationships and potential mediators, and each mediation pathway was tested using the model-based causal mediation framework implemented in the *mediation* package in R.^14^ Where mediation was detected, the authors fitted appropriate regression models to avoid misinterpreting adjusted coefficients as causal effects, see Supplementary Text S5 (Appendix Page 9). R version 4.5.1 was used for all statistical analysis.

### Ethics approval

The study protocol was approved by the Human Research Ethics Committee of the University of Sydney (ID: 2020/818) and the Australian Institute of Aboriginal and Torres Strait Islander Studies (ID: EO303-20211008). Additional ethics approvals were obtained from state-based Indigenous human research ethics organisations. The AEEHS was conducted in accordance with the tenets of the Declaration of Helsinki with signed written consent provided by all participants. The study was also conducted in accordance with the CONSIDER statement, AIATSIS Code of Ethics for Aboriginal and Torres Strait Islander Research, and other guidelines for research in Indigenous communities. Supplementary Text S1 and Supplementary Fig. S2 (Appendix Pages 2–6).^15^

### Role of the funding source

The funders of the study had no role in study design, data collection, data analysis, data interpretation, or writing of the manuscript.

## Results

In total, 617 Indigenous Australians (53.2% female versus 46.8% male; mean age [Standard Deviation, SD] 63.8 [10.6] years), and 3902 non-Indigenous Australians (54.8% female versus 45.2% male; mean age 70.5 [9.8] years) were examined. All participants underwent the eye examination, and 3573/4519 (79.1%) participants completed both the eye and ear examinations.

A study flowchart detailing participation at each of the study stages and reasons advised for declining eye and/or ear examination are provided in Supplementary Fig. S3 (Appendix Page 7). Compared to non-Indigenous participants, Indigenous participants were younger, less likely to have completed high school, have private health insurance, own a home, or attend an eye examination in the last 12 months, and more likely to have diabetes (2-fold), hypertension, hypercholesterolaemia and live in regional/remote areas (Table 1).

**Table 1:** Demographics of the AEEHS sample.

	Indigenous n (%)	Non-Indigenous n (%)	Total n
Participants	617 (13.6)	3902 (86.4)	4519
Age Groups (years)			
50–59	211 (34.2)	582 (14.9)	793
60–69	238 (38.6)	1188 (30.5)	1426
70–79	117 (19.0)	1389 (35.6)	1506
80+	51 (8.3)	743 (19.0)	794
Mean age (SD)	63.8 (10.6)	70.5 (9.8)	69.6 (10.2)
Gender			
Male	289 (46.8)	1765 (45.2)	2054
Female	328 (53.2)	2137 (54.8)	2465
Remoteness			
Major Cities	46 (7.5)	2460 (63.0)	2506
Inner Regional	99 (16.0)	544 (13.9)	643
Outer Regional	188 (30.5)	654 (16.8)	842
Remote/Very Remote	284 (46.0)	244 (6.3)	528
Highest education level			
Below high school	317 (51.4)	1212 (31.1)	1529
High school	29 (4.7)	446 (11.4)	475
Tertiary	271 (43.9)	2244 (57.5)	2515
Owns home			
Yes	307 (49.8)	3167 (81.2)	3474
No	310 (50.2)	735 (18.8)	1045
Private health insurance			
Yes	150 (24.3)	2238 (57.4)	2388
No	467 (75.7)	1664 (42.6)	2131
Lives alone			
Yes	202 (32.7)	1092 (28.0)	1294
No	415 (67.3)	2810 (72.0)	3225
Smoking status			
Never smoked	270 (43.8)	2701 (69.2)	2971
Current smoker	162 (26.2)	214 (5.5)	376
Ever smoked	185 (30.0)	987 (25.3)	1172
Body Mass Index			
Not obese (<30 kg/m^2^)	266 (43.1)	961 (24.6)	1227
Obese (≥30 kg/m^2^)	351 (56.9)	2941 (75.4)	3292
Self-rated health			
Poor/Fair	216 (35.0)	836 (21.4)	1052
Good/Excellent	398 (65.0)	3063 (78.5)	3461
Not known	3	3	6
Diabetes			
Yes	213 (34.5)	583 (14.9)	796
No	404 (65.5)	3319 (85.1)	3723
History of cardiovascular disease			
Yes	111 (18.0)	396 (10.1)	507
No	506 (82.0)	3506 (89.9)	4012
History of cerebrovascular disease			
Yes	42 (6.8)	194 (5.0)	236
No	575 (93.2)	3708 (95.0)	4283
Hypertension			
Yes	332 (53.8)	1786 (45.8)	2118
No	285 (46.2)	2116 (54.2)	2401
Had eye exam in last 12 months			
Yes	309 (50.1)	2611 (66.9)	2920
No	308 (49.9)	1291 (33.1)	1599

SD refers to Standard Deviation.

Table 2 shows that for the >25 dB HL category, the overall crude prevalence of DSI was 3.3%, and for the more severe (>40 dB HL) HI category, crude prevalence was 1.6%. A larger proportion of Indigenous participants had DSI than non-Indigenous participants (6.3% versus 2.9%, p < 0.001, for the >25 dB HL category; and 3.3% versus 1.3%, p = 0.004 for the >40 dB HL category). While the difference in prevalence rates was non-significant after age-standardization to the 2021 Australian population stratified by Indigenous status (Table 2), reflecting variations in the age distribution and age-specific prevalence of DSI (for the >25 dB HL category) between Indigenous and non-Indigenous populations; there still appears to be a noteworthy difference in prevalence rates that could be clinically meaningful: 5.2%, 95% CI (3.3–7.9) for Indigenous and 2.8%, 95% (2.2–3.4) for non-Indigenous participants (p = 0.047). Further, when adjusted for age and remoteness, the prevalence of any DSI was 5.5% (95% CI: 3.2–9.4) for Indigenous, 2.7% (95% CI:2.1–3.4%) for non-Indigenous Australians, and 2.7% (95% CI: 2.2–3.4) for the overall sample, Supplementary Table S4 (Appendix Page 8). In our study, the Indigenous subgroup had a markedly different demographic composition, particularly with respect to age and remoteness, compared with the non-Indigenous subgroup. After standardization, the weighting applied to each subgroup differed from the weighting contributing to the overall adjusted estimate, resulting in the overall prevalence estimate being closer to the non-Indigenous estimate and, in some instances, lower than both subgroup-specific adjusted estimates.

**Table 2:** Prevalence of dual sensory impairment prevalence in the AEEHS, among Indigenous and non-Indigenous participants, by level of hearing impairment, before and after age standardisation to the Australian population.

	Indigenous		Non-Indigenous		Total		p-value
	N	% (95% CI)	N	% (95% CI)	N	% (95% CI)	
Crude prevalence							
Bilateral vision impairment + any bilateral hearing impairment (definition: >25 dB HL)							
Yes	29	6.3 (4.3–9.0)	90	2.9 (2.3–3.6)	119	3.3 (2.8–4.0)	<0.001
Bilateral vision impairment + moderate or worse bilateral hearing impairment (definition: >40 dB HL)							
Yes	15	3.3 (1.9–5.4)	42	1.3 (1.0–1.8)	57	1.6 (1.2–2.1)	0.004
Age-standardized prevalence							
Bilateral vision impairment + any bilateral hearing impairment (definition: >25 dB HL)							
Yes	29	5.2 (3.3–7.9)	90	2.8 (2.2–3.4)	119	2.5 (2–3.1)	0.047
Bilateral vision impairment + moderate or worse bilateral hearing impairment (definition: >40 dB HL)							
Yes	15	2.8 (1.5–5.2)	42	1.3 (0.9–1.8)	57	1.3 (0.9–1.7)	0.115

CI = confidence interval, dB HL = decibels Hearing Level.

**Fig. 1:**
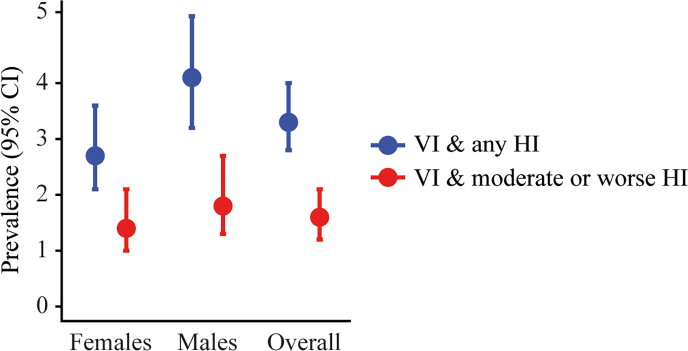
Crude prevalence of dual sensory impairment by gender.

Table 3 shows crude DSI prevalence by age group and Indigenous status. In the overall population, the prevalence of DSI for the >25 dB HL and >40 dB HL categories, increased by around 13- and 9-fold (p < 0.0001), respectively, between ages 50 to 80+ years. The higher prevalence of DSI among Indigenous compared to non-Indigenous persons was particularly apparent among those aged under 70 years, where it was at least 3-fold higher for both the >25 and >40 dB HL categories. A statistically significant trend (p < 0.0001) for increasing prevalence of DSI for both hearing loss categories was observed with increasing age in non-Indigenous participants. Overall, men had a higher prevalence of DSI (for the >25 dB HL category) than women (Fig. 1). This difference was not observed for the prevalence for the >40 dB HL category.

**Table 3:** Crude prevalence of dual sensory impairment, by age group and Indigeneity.

Age group	Indigenous		Non-Indigenous		Total	
	N	% (95% CI)	N	% (95% CI)	N	% (95% CI)
Bilateral Vision Impairment + Any Bilateral Hearing Impairment (Definition: >25 dB HL)						
50–59	4	2.9 (0.9–7.7)	0		4	0.7 (0.3–1.8)
60–69	12	6.6 (3.6–11.5)	4	0.4 (0.1–1.2)	16	1.4 (0.9–2.4)
70–79	9	9.2 (4.5–17.2)	28	2.5 (1.7–3.7)	37	3.0 (2.2–4.2)
80+	4	9.5 (3.1–23.5)	58	9.1 (7.1–11.7)	62	9.1 (7.1–11.6)
p-trend		0.03		<0.0001		<0.0001
Bilateral Vision Impairment + Moderate or Worse Bilateral Hearing Impairment (Definition: >40 dB HL)						
50–59	3	2.2 (0.6–6.7)	0		3	0.5 (0.2–1.5)
60–69	6	3.3 (1.3–7.4)	3	0.3 (0.1–1)	9	0.8 (0.4–1.6)
70–79	3	3.1 (0.8–9.3)	11	1.0 (0.5–1.8)	14	1.2 (0.7–2)
80+	3	7.1 (1.9–20.6)	28	4.4 (3–6.4)	31	4.6 (3.2–6.5)
p-trend		0.20		<0.0001		<0.0001

CI = confidence interval, dB HL = decibels Hearing Level.

After adjustment in multivariable analysis, age per 10 years and not having private health insurance were independently and significantly associated with a greater likelihood of DSI (for the >25 dB HL category; Table 4) in both Indigenous and non-Indigenous participants. In terms of PAR, not having private insurance accounted for 34.0% and 86.2% of the total DSI (for >25 dB HL category) burden in non-Indigenous and Indigenous adults, respectively. However, these PAR estimates should be interpreted cautiously, as they are model-based and reflect both the prevalence of the exposure and its association with DSI, rather than indicating that the equivalent proportion of cases would be eliminated through changes in private insurance status alone.

**Table 4:** Multivariable adjusted logistic regression model for risk factors associated with bilateral vision impairment and any bilateral hearing impairment (>25 dB HL) among Indigenous and non-Indigenous participants.

Risk factors	Non-Indigenous			Indigenous		
	Prevalence	OR (95%CI)	p value	Prevalence	OR (95%CI)	p value
Age		1.16 (1.13–1.2)	0.00		1.06 (1.01–1.11)	0.02
Male sex	3.5 (2.6–4.7)	1.36 (0.86–2.17)	0.19	7.4 (4.5–11.8)	1.28 (0.57–2.95)	0.55
Education						
High school	3.5 (1.9–6.2)	1.5 (0.72–2.96)	0.26	9.1 (1.6–30.6)	1.11 (0.16–4.74)	0.90
Tertiary	2.1 (1.5–2.9)	1.01 (0.61–1.66)	0.98	2.2 (0.7–5.9)	0.39 (0.11–1.09)	0.10
Smoking status						
Current smoker	2.6 (0.8–7.0)	1.22 (0.34–3.42)	0.73	6.1 (2.7–12.7)	0.98 (0.33–2.81)	0.97
Ever smoker	3.1 (2.0–4.6)	0.91 (0.53–1.53)	0.74	6.4 (3.1–12.1)	0.91 (0.35–2.3)	0.85
Type 2 diabetes	4.3 (2.6–6.8)	1.34 (0.73–2.32)	0.32	8.0 (4.5–13.5)	1.3 (0.55–3.03)	0.54
Hypertension	3.6 (2.7–4.7)	0.84 (0.53–1.34)	0.46	7.6 (4.7–11.8)	1.03 (0.44–2.54)	0.95
Cardiovascular disease	0.6 (0.0–4.1)	0.11 (0.01–0.5)	0.03	11.8 (3.8–28.4)	1.7 (0.44–5.38)	0.40
Excellent/Good self-reported health	2.8 (2.2–3.6)	1.13 (0.66–2.01)	0.66	6.8 (4.3–10.5)	2.08 (0.89–5.25)	0.10
Not having private insurance	4.1 (3.1–5.4)	2.27 (1.44–3.6)	0.00	8.2 (5.6–11.7)	9.39 (1.84–172)	0.03
Regionality						
Inner Regional	0.0 (0.0–1.4)	0.00 (0.00–1.76)	0.98	7.1 (2.3–18.1)	2.3 (0.29–48.46)	0.48
Outer Regional	3.3 (2.1–5.1)	1.00 (0.57–1.7)	1.00	4.0 (1.8–8.4)	1.46 (0.23–28.93)	0.74
Remote	5.9 (3.0–10.9)	2.87 (1.27–5.96)	0.01	8.7 (5.3–13.8)	3.43 (0.57–66.67)	0.27

CI = confidence interval, dB HL = decibels Hearing Level; OR = odds ratio.

In non-Indigenous adults, living in remote areas versus in a major city was associated with greater odds of DSI (for >25 dB HL category): OR 2.87 (95% CI 1.27–5.96) and the PAR for this was 9.3% of the total DSI burden. Table 5 shows that among non-Indigenous adults increasing age (OR 1.17; 95% CI: 1.13–1.23) and not having private insurance (OR 2.26; 95% CI: 1.17–4.43) were significantly and independently associated with prevalence of DSI (for the >40 dB HL category). Not having private insurance was responsible for 33.9% of the DSI (for >40 dB HL category) burden in non-Indigenous Australians; and these findings suggest that socioeconomic disadvantage and limited access to affordable services are likely to be a possible underlying explanation for these observed associations. No significant risk factors for prevalence of DSI (for >40 dB HL category) was observed in Indigenous adults (Table 5).

**Table 5:** Multivariable adjusted logistic regression model for risk factors associated with bilateral vision impairment and any bilateral hearing impairment (>40 dB HL) among Indigenous and non-Indigenous participants.

Risk factors	Non-indigenous			Indigenous		
	Prevalence	OR (95%CI)	p	Prevalence	OR (95%CI)	p value
Age		1.17 (1.13–1.23)	0.00		1.04 (0.98–1.11)	0.20
Male sex	1.5 (1.0–2.4)	1.24 (0.63–2.41)	0.53	3.5 (1.6–7.0)	1.02 (0.33–3.21)	0.97
Education						
High school	1.7 (0.7–3.9)	2.1 (0.71–5.54)	0.15	9.1 (1.6–30.6)	2.88 (0.36–15.77)	0.25
Tertiary	1.1 (0.7–1.7)	1.43 (0.69–2.98)	0.34	0.6 (0.0–3.5)	0.19 (0.01–1.05)	0.12
Smoking status						
Current smoker	0.7 (0.0–4.2)	0.55 (0.03–3.09)	0.58	1.8 (0.3–6.8)	0.4 (0.05–1.94)	0.29
Ever smoker	1.3 (0.7–2.4)	0.8 (0.35–1.68)	0.58	3.5 (1.3–8.5)	0.77 (0.21–2.57)	0.68
Type 2 diabetes	2.4 (1.2–4.5)	1.53 (0.67–3.23)	0.29	5.5 (2.7–10.5)	1.97 (0.62–6.69)	0.26
Hypertension	1.8 (1.2–2.6)	0.9 (0.46–1.8)	0.77	3.6 (1.8–6.9)	0.74 (0.23–2.55)	0.62
Cardiovascular disease	0.0 (0.0–3.0)	0 (0–7.58 × 10^15^)	0.99	2.9 (0.2–17.1)	0.82 (0.04–5.02)	0.86
Excellent/Good self-reported health	1.1 (0.7–1.6)	0.5 (0.26–1.01)	0.05	3.4 (1.7–6.4)	1.67 (0.53–6)	0.40
Not having private insurance	2.0 (1.3–2.9)	2.26 (1.17–4.43)	0.02	4.4 (2.6–7.3)	0 (0–1.77 × 10^211^)	0.99
Regionality						
Inner Regional	0.0 (0.0–1.4)	0 (0–9.12 × 10^7^)	0.99	3.6 (0.6–13.4)	1.15 (0.09–29.28)	0.92
Outer Regional	1.1 (0.5–2.4)	0.67 (0.26–1.5)	0.36	0.6 (0.0–3.6)	0.22 (0.01–6.37)	0.31
Remote	1.8 (0.5–5.5)	1.63 (0.36–5.19)	0.46	5.6 (3.0–10.1)	2.23 (0.3–48.92)	0.50

CI = confidence interval, dB HL = decibels Hearing Level; OR = odds ratio.

We assessed potential misinterpretation of multivariable coefficients (the ‘Table 2 fallacy’). In DAG 1 (exposure: age; mediator: private insurance; outcome: dual sensory impairment), no significant mediation was observed, suggesting minimal risk of bias. In DAG 2 (exposure: diabetes; mediator: private insurance; outcome: dual sensory impairment), a significant indirect effect was identified (p < 0.001), accounting for 21% and 15% of the total effect for DSI defined as hearing loss >25 dB HL and >40 dB HL, respectively. However, comparisons of models with and without the mediator showed only small changes in effect estimates, with no meaningful impact on statistical significance or overall interpretation. These findings indicate that, despite some mediation in the diabetes pathway, the estimated associations can be interpreted as total effects without substantial bias from the ‘Table 2 fallacy’; see Supplementary Text S5 (Appendix Page 9).

## Discussion

This nationwide-first survey found that one in 40 Australian adults aged over 50 have DSI (any bilateral VI and HI). This is in agreement with a recent review which reported international prevalence rates of between 1.6% and 18.2% in population-based studies of adults aged 50+ years.^1^ The Blue Mountains Eye Study or BMES (a landmark sensory loss study of older Australians) conducted over 20 years ago reported an overall prevalence of objectively measured DSI of 6.03% in adults aged 55+ years^16^; around 2-fold higher than the age-standardized prevalence rate observed in the current survey. This comparative reduction in prevalence of DSI may reflect improvements in eye and hearing health services over time e.g., better management of chronic eye diseases (e.g., age-related macular degeneration, diabetic retinopathy, glaucoma) through modern treatments such as intravitreal therapies.

For the overall Australian population and in non-Indigenous adults particularly, the prevalence of DSI rose steeply with age, with the greatest prevalence of DSI (for any HI) of 9.1% observed in adults aged 80+. This concurs with data from the BMES showing that DSI most likely affects the oldest of the old,^16^ and also aligns with age-related trends reported worldwide, where DSI has been reported more commonly among people aged 80 years or older.^1^ Recently, 2579 participants aged 71+ years from the US National Health Aging Trends Study (NHATS)^17^ also demonstrated that the prevalence of DSI increased with age, although substantially higher prevalence rates (versus our study) of 15% was observed among those aged 71–75 years and 46% for those 85–90 years. Overall, in our study males had a higher prevalence of DSI than women for the >25 dB HL category, though sex differences diminished with more severe hearing loss in Australians aged 50+. This observation also concurs with US data from the NHATS.^17^ These trends are expected to continue with the ageing population and emphasise the importance of routine sensory health care in older adults, particularly males.^3^

This study provides novel epidemiological data on the prevalence of DSI in Indigenous Australians. The overall age-standardised prevalence of DSI (for both >25 and >40 dB HL categories) were relatively similar between Indigenous and non-Indigenous participants. However, there was a consistent trend for DSI to be more prevalent in Indigenous compared to non-Indigenous adults in the 50–79 age groups. For example, the 16.5- and 11-fold greater rate of DSI for the >25 and >40 dB HL categories, respectively, observed in Indigenous adults aged 60–69 years compared to their non-Indigenous peers is concerning. Global and Australian data on the prevalence of DSI in Indigenous adults are lacking, but indicators suggest it is likely higher due to the increased prevalence of HI and VI in the Indigenous population. Namely, HI is highly prevalent, with earlier onset in Indigenous Australians due to factors like chronic middle ear disease,^18^ and VI is also much more common, with a previous survey showing rates of any VI were 2.8 times higher in Indigenous adults over 40 compared to non-Indigenous Australians.^19^

The relatively high prevalence of DSI observed in both Indigenous and non-Indigenous Australians, impacting almost 1 in 10 aged 80 years and older, indicates that this condition should be considered in the delivery of stand-alone HI and VI services. Namely, for adults with a single sensory loss, vision can sometimes compensate for HI and auditory cues can sometimes compensate for VI. However, the combination of both impairments together can have compounded impacts on social participation, mental health and quality of life, as well as impacting approaches to care and rehabilitation.^20^ Epidemiological data from this study reinforces the importance of professionals working in the vision and hearing health sectors recognizing that many of their older clients may have DSI. Active case-finding among older adults with single sensory loss may help identify and connect people living with DSI to appropriate services and supports.^3^ Often service providers with training in one of these areas do not have knowledge about the other, and so may not be able to adequately assess or address the impact of the second sensory impairment with regard to rehabilitation and treatments.^6^^,^^16^ Therefore, greater efforts towards cross-sector collaboration are required to support the unique needs of this growing population of older adults with DSI, in a holistic and integrated manner.^6^^,^^16^

This study has also identified novel risk factors that could be targeted to minimize the likelihood of developing DSI, including lack of private insurance and remote/very remote residence. These data are important from both a practice and public health perspective because, while treatments and interventions (e.g., hearing aids and cataracts surgery) are available to address some forms of DSI,^2^^,^^21^ these may not be accessible or timely, specifically in the higher risk groups. Additionally, efficacious prevention and management strategies is warranted, not only because of the high prevalence rates, but also because combined sensory impairments are major causes of disability.^2^^,^^21^

Namely, lack of private insurance was a significant and independent predictor of DSI (for >25 dB HL) in both non-Indigenous and Indigenous adults. The high proportion of burden attributable to absence of private insurance underscores the critical influence of socioeconomic disadvantage and limited access to affordable hearing and vision services in the Australian population. In non-Indigenous adults, residence in remote/very remote regions accounts for 8.8% of the total DSI burden (for >25 dB HL) in Australia, respectively. Similarly, the recent China Health and Retirement Longitudinal Study (CHARLS),^22^ a nationally representative biennial survey of adults aged 45+ across 28 provinces found that rural residence was a stable risk factor for prevalence of self-reported DSI. Some potential reasons for this observation in both Australia and China, are that people in rural and remote areas face ongoing barriers to specialized eye and ear care, with distance, cost, and workforce shortages limiting access and delaying early diagnosis and treatment of sensory loss.^22^ Further, there is a well-known lack of integrated care access in rural communities in Australia, likely limiting the diagnosis and management for those with DSI.^23^ These findings highlight substantial and preventable inequities in sensory health.

From a practice and public health perspective, these study findings suggest that interventions which expand equitable, community-based, and culturally safe hearing and vision services could substantially reduce the national DSI burden. Policy responses should prioritize integrating screening and rehabilitation within primary and aged-care settings, strengthening outreach to rural and Indigenous communities, and improving affordability through publicly funded or subsidized care models to reduce preventable sensory disability and promote healthy ageing. Clinically, these results reinforce the need for targeted and person-centered screening, early intervention, and rehabilitation strategies for remote/very remote and Indigenous populations.

Strengths of the AEEHS include the multistage sampling methodology that was designed to ensure sufficient Indigenous representation, utilization of advanced ophthalmic and audiological imaging, and concurrent eye and ear examinations. Recruitment from a range of geographical sites uncovered the major effect of remoteness as a risk factor for DSI, which could have been missed if fewer sites were sampled. Limitations include the study not being able to distinguish between congenital and acquired sensory impairments, which may have led to misclassification when interpreting DSI. For instance, when one impairment is congenital and the other acquired, the condition may not be entirely preventable through public health measures. Further, our case definition was based on presenting distance visual acuity in the better eye and unaided hearing thresholds (PTA) in the better ear and therefore, did not assess binocular visual function (e.g., stereopsis) or binaural hearing function (e.g., spatial hearing/speech-in-noise). Hence, we may have underestimated the functional impact of DSI in daily life and misclassified some participants whose primary difficulties relate to binocular or binaural processing rather than monocular acuity or pure-tone thresholds. Additionally, given that our AEEHS definition of DSI is an objectively measured classification (visual acuity plus audiometric thresholds), this is important in interpreting the implications of this study because eligibility and supports through the Australian National Disability Insurance Scheme (NDIS), are determined through an individualised and functional process (often requiring specialist documentation), so not all people who meet AEEHS DSI criteria (objectively measured classification) would necessarily meet NDIS access criteria, and conversely some individuals with significant access needs may not be identified using threshold-based DSI cut-points. Finally, the AEEHS is a cross-sectional study, and we cannot establish causal relationships between the factors identified and DSI prevalence.

In summary, DSI represents a significant and under-recognized public health challenge in Australia, particularly among the oldest age groups. This nationally representative survey has addressed existing knowledge gaps, by demonstrating that DSI prevalence increases sharply with age and disproportionately affects Indigenous Australians, those living in remote regions, and individuals without private health insurance. These findings reveal the compounded influence of biological ageing, socioeconomic disadvantage and structural inequities on sensory health. As the population ages, the burden of coexisting hearing and vision loss will escalate, amplifying impacts on independence, mental health, and quality of life. Addressing DSI therefore requires an integrated, equity-focused approach to sensory care, one that bridges hearing and vision services, strengthens access in underserved communities, and embeds routine dual screening within primary and aged care. Proactive policy and service responses are essential to mitigate the growing sensory health gap and support healthy ageing for all Australians.

## Contributors

BG–conceptualisation, data curation, funding acquisition, investigation, methodology, resources, supervision, writing (original draft), and writing (review & editing). RK–conceptualisation, data curation, formal analysis, investigation, methodology, project administration, resources, supervision, visualisation, and writing (review & editing). OM–conceptualisation, data curation, formal analysis, investigation, methodology, resources, and writing (review & editing). GLow—formal analysis, methodology, validation, visualisation, writing (original draft), and writing (review & editing). YK–data curation, investigation and writing (review & editing). CW–conceptualisation, funding acquisition, investigation, methodology, supervision and writing (review & editing). MI–data curation, investigation and writing (review & editing). TF–conceptualisation, funding acquisition, investigation, methodology, supervision and writing (review & editing). DT–investigation and writing (review & editing). EY–data curation and writing (review & editing). JN–investigation, methodology, supervision and writing (review & editing). AT–investigation and writing (review & editing). LK–conceptualisation, data curation, funding acquisition, investigation, methodology, resources, supervision and writing (review & editing). GL–conceptualisation, data curation, formal analysis, funding acquisition, investigation, methodology, project administration, resources, supervision, and writing (review & editing). PM–conceptualisation, data curation, formal analysis, funding acquisition, investigation, methodology, project administration, resources, supervision, and writing (review & editing). BG had final responsibility for the decision to submit for publication. All authors had full access to all the data in the study. All authors read and approved the final version of the manuscript. BG, RK, Glow, and GL directly accessed and verified the underlying data reported in the manuscript.

## Data sharing statement

Data underlying the results in the study are considered highly sensitive as they involve the health status of Indigenous individuals, as well as non-Indigenous individuals in vulnerable situations. Even when de-identified there is potential for harm and this will be considered when assessing requests for data sharing. These can be considered if the proposal has been approved by an independent review committee and the aims cannot be achieved through any other channels. The proposal will be considered by the AEEHS committee, if received between 12 and 36 months of publication, through contact with the corresponding author. The study protocol and procedures are a different issue and can be shared if a proposal has been approved by a learned intermediary, a written proposal is provided, and the corresponding author is contacted directly.

## Declaration of interests

Angus Turner is a Board Member of the Lions Eye Institute. Lisa Keay has received support from the WHO to attend Strategic and Technical Advisory Committee meetings for the SPEC2030 initiative. She also is on the Steering committee for an RCT of refractive error correction and motorcycle safety in Vietnam (the STABLE trial). Finally, she is a member of Vision 2020 Australia, representing the School of Optometry and Vision Science at University of New South Wales. Other authors report no conflicts of interests.
