# Supplementary material for: Prevalence of dual sensory impairment in Australia: a nationally representative population-based survey of Indigenous and non-Indigenous Australians
Source: Lancet Reg Health West Pac. 2026 Jun 5;71:101897. doi: 10.1016/j.lanwpc.2026.101897 (PMC13266250; doi:10.1016/j.lanwpc.2026.101897)
Supplement: Supplementary File [file mmc1.docx]

**Prevalence of dual sensory impairment in Australia: a nationally representative population-based survey of Indigenous and non-Indigenous Australians**

**Supplementary File**

**Table of Contents**

Supplementary Text S1. CONSIDER checklist for the Australian Eye and Ear Health Survey (AEEHS) 2

Supplementary Figure S2. Aboriginal and Torres Strait Islander Quality Appraisal Tool6

Supplementary Figure S3. Flow chart of participation in the AEEHS 7

Supplementary Table S4. Prevalence of Dual Sensory Impairment (Age & Remoteness-Standardised) to the projected 2023 Australian Population data provided by the Australian Institute of Health and Welfare8

Supplementary Text S5. Mediation analysis output and Directed Acyclic Graphs 9

References14

**Supplementary Text S1. CONSIDER checklist for the Australian Eye and Ear Health Survey (AEEHS)^1^**
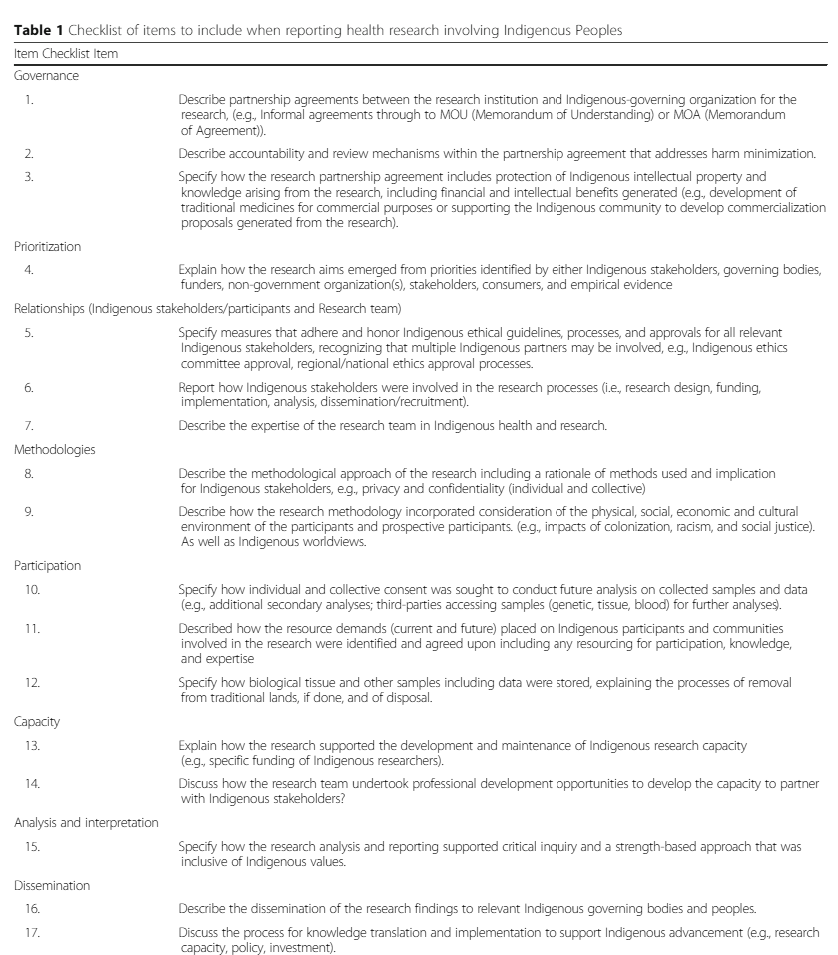


| **Governance** | **Describe partnership agreements between the research institution and Indigenous-governing organisation for the research**  Partnerships underpinning the AEEHS included Indigenous co-investigators, numerous Aboriginal Community Controlled Health Organisations (ACCHOs), the Brien Holden Foundation, and community representatives. Aboriginal and Torres Strait Islander governance and advisor input were embedded from study conception through implementation. This engagement informed study design, recruitment, and conduct to ensure culturally safe approaches. |
| --- | --- |
|  | **Describe accountability and review mechanisms with the partnership agreement that addresses harm minimisation**  Ongoing consultation with the above-mentioned partnerships provided oversight and accountability throughout the study, including guidance on culturally appropriate engagement and harm minimisation. Ethics approvals were obtained from the University of Sydney, Australian Institute of Aboriginal and Torres Strait Islander Studies, which is the national Indigenous ethics committee of Australia, and relevant state-based Indigenous ethics committees. The ethics committees provided specific requirement for the conduct, storage and reporting of sensitive data. |
|  | **Specify how the research partnership agreement includes protection of indigenous intellectual property and knowledge arising from the research including financial and intellectual benefits generated**  Data governance processes were aligned with principles of Indigenous data sovereignty, including protection of Indigenous knowledge and appropriate management of intellectual and community benefits arising from the research, in accordance with guidance from Indigenous ethics bodies and partner organisations. |
| **Prioritization** | **Explain how the research aims emerged from priorities identified by either Indigenous stakeholders, governing bodies, funders, non-government organisations, stakeholders, consumers and empirical evidence**  The aims of the AEEHS were informed by gaps in nationally representative data on vision and hearing impairment and priorities identified by Indigenous stakeholders. These priorities aligned with national policy and funding agendas to reduced health disparities and with empirical evidence demonstrating a disproportionate burden of unmet need among Indigenous Australians. This shaped the study’s focus on prevalence, unmet need and service access. |
| **Relationships** | **Specify measures that adhere and honour indigenous ethical guidelines, processes and approvals for all relevant indigenous stakeholders recognising that multiple indigenous partners may be involved e.g. Indigenous ethics committee approval, regional/national ethics approval processes**  The AEEHS adhered to Indigenous ethical guidelines through approvals from the Australian Institute of Aboriginal and Torres Strait Islander Studies (AIATSIS) and the relevant state-based Indigenous ethics committees, recognising jurisdictional requirements across multiple regions. The study team also consulted with local ACCHOs and First Nations elders in each site for their guidance to ensure that the study was conducted in a culturally appropriate manner. These processes guided culturally appropriate conduct, governance and reporting, ensuring respect for Indigenous knowledge, community expectations, and ethical standards across all participating sites. |
|  | **Report how indigenous stakeholders were involved in the research processes i.e. research design, funding, implementation, analysis, dissemination and recruitment**  Aboriginal and Torres Strait Islander stakeholders, including our co-investigators, were involved across all stages of the AEEHS including study design, recruitment strategies, implementation and dissemination planning. Engagement with Indigenous stakeholders informed culturally appropriate approaches and supported participation, however involvement in funding decisions and formal analysis processes was limited. |
|  | **Describe the expertise of the research team in indigenous health and research**  The research team included Aboriginal and Torres Strait Islander investigators/ collaborators with expertise in Indigenous health, community engagement, and culturally responsive research. This was complemented by the overall team’s multidisciplinary expertise in epidemiology, audiology, ophthalmology, and population health, supporting the conduct and interpretation of the study within appropriate cultural and clinical contexts. |
| **Methodologies** | **Describe the methodological approach of the research including a rationale of methods used and implication for indigenous stakeholders e.g. privacy and confidentiality**  The AEEHS employed a cross-sectional nationally representative design using stratified multi-stage cluster sampling across 30 sites. Oversampling of Aboriginal and Torres Strait Islander participants was implemented through stratification by Indigenous population density to ensure sufficient statistical power and precision for subgroup analyses, addressing historical under-representation. Ethical approvals guided strict protocols for privacy, confidentiality and secure data management, minimising risk and protecting sensitive data. |
|  | **Describe how the research methodology incorporated consideration of the physical, social, economic and cultural environment of the participants and prospective participants as well as indigenous world views.**  The methodology incorporated geographic, social and cultural context through sampling across urban, regional and remote settings and collaboration with indigenous stakeholders to support culturally appropriate engagement. Engagement with communities supported alignment with Indigenous perspectives on health, participation and access. |
| **Participation** | **Specify how individual and collective consent was sought to conduct future analysis on collected samples and data**  Written informed consent was obtained from all participants prior to data collection, including consent for future analyses of de-identified data. Consent processes were guided by Indigenous ethics approvals and designed to ensure cultural appropriateness and clarity regarding data use. Where relevant, engagement with community organisations supported broader awareness of study aims and potential future use of data, consistent with principals of Indigenous data governance. |
|  | **Describe how the resource demands placed on indigenous participants and communities involved in the research were identified and agreed upon including any resourcing for participation, knowledge and expertise.**  The resource demands placed on Indigenous participants and communities were considered through consultation with ACCHOs and community representatives during study planning and implementation. Study procedures were designed to minimise participant burden, including localised data collection and streamlined assessments. Engagement with community organisations recognised the contribution of local knowledge and expertise, with support provided for participation and facilitation of recruitment and study delivery |
|  | **Specify how biological tissue and other samples including data were stored, explaining the processes of removal from traditional lands if done and of disposal**  Data were securely stored in accordance with institutional and Indigenous ethics requirements, including controlled access and de-identification procedures. No biological tissue samples were stored as part of the AEEHS. |
| **Capacity** | **Explain how the research supported the development and maintenance of indigenous research capacity e.g. specific funding of indigenous researchers**  The AEEHS supported Indigenous research capacity through involvement of Aboriginal and Torres Strait Islander investigators, data collectors, and partnerships with ACCHOs across study design and implementation. These collaborations facilitated knowledge exchange and participation in large-scale epidemiological research. |
|  | **Discuss how the research team undertook professional development opportunities to develop the capacity to partner with indigenous stakeholders**  The research team developed capacity to partner with Indigenous stakeholders through ongoing engagement with indigenous investigators, Elders and community organisations. This supported understanding of cultural protocols and ethical requirements and community priorities, in turn strengthening culturally responsive research practices and informing study conduct. |
| **Analysis and interpretation** | **Specify how the research analysis and reporting supported critical inquiry and a strength-based approach that was inclusive of indigenous values**  Analysis and reporting were undertaken with consideration of social and structural determinants of health, supporting critical interpretation beyond individual-level deficit framing. Findings were contextualised within access to care and systemic inequities and reported using a strength-based approach that emphasises opportunities for improved service delivery consistent with Indigenous values and perspectives. |
| **Dissemination** | **Describe the dissemination of the research findings to relevant indigenous governing bodies and peoples**  Findings have begun to be disseminated to Aboriginal and Torres Strait Islander stakeholders, including ACCHOs, participating communities and relevant governing bodies through accessible reports, presentations and stakeholder engagement. Dissemination approaches have been informed by prior consultation to ensure results are communicated in culturally appropriate and meaningful formats. |
|  | **Discuss the process for knowledge translation and implementation to support indigenous advancement e.g. research capacity, policy, investment.**  Knowledge translation will focus on informing policy, service planning and investment to improve equitable access to eye and hearing care. Findings are intended to support Indigenous-led health services, guide resource allocation, and strengthen evidence for targeted interventions. In turn contributing to improved health outcomes and ongoing capacity development within Indigenous health systems. |

## **
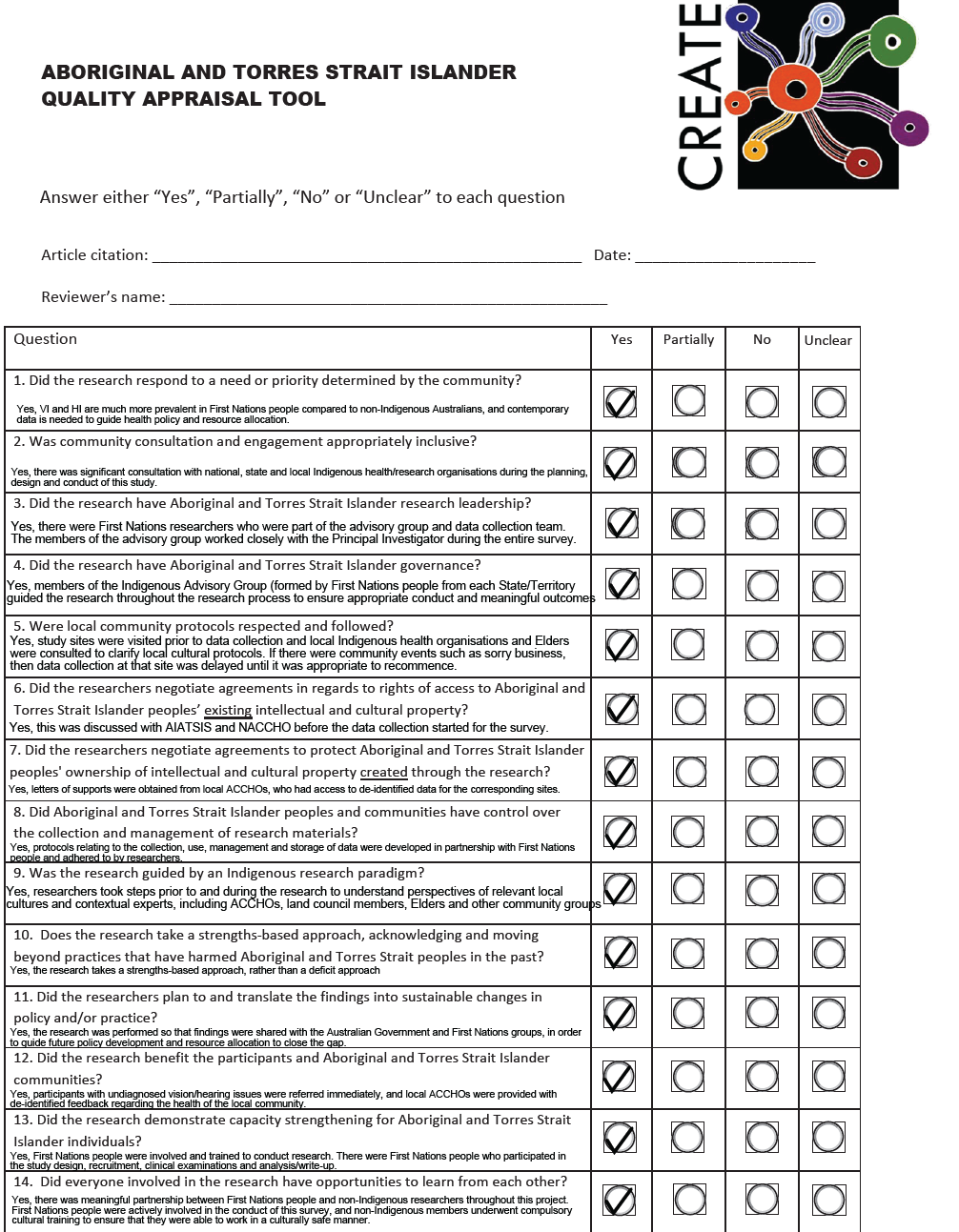
Supplementary Figure S2. Aboriginal and Torres Strait Islander Quality Appraisal Tool^2^**

## **Supplementary Figure S3. Flow chart of participation in the AEEHS**


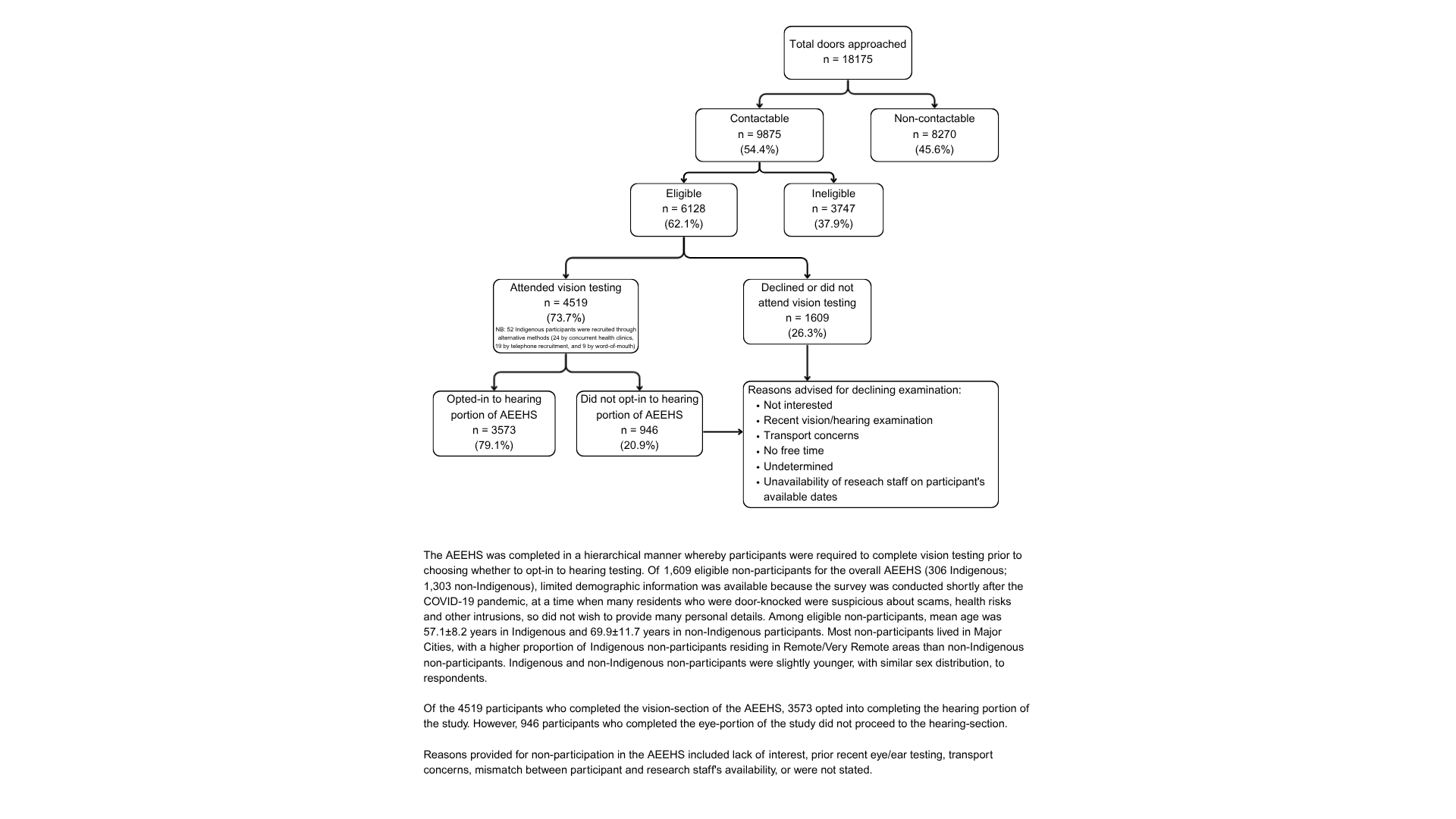


## **Supplementary Table S4. Prevalence of Dual Sensory Impairment (Age & Remoteness-Standardised) to the projected 2023 Australian Population data provided by the Australian Institute of Health and Welfare**

|  | **Indigenous, % (95% CI)** | **Non-Indigenous, % (95% CI)** | **Total, % (95% CI)** |
| --- | --- | --- | --- |
| Bilateral Vision Impairment + Any Bilateral Hearing Impairment (>25 dB HL) | 5.5 (3.2-9.4) | 2.7 (2.1-3.4) | 2.7 (2.2-3.4) |
| Bilateral Vision Impairment + Moderate or Worse Bilateral Hearing Impairment (>40 dB HL) | 3.4 (1.5-7.4) | 1.8 (1.3-2.5) | 1.5 (1.1-2.1) |

CI = confidence interval; dB HL=decibels Hearing Level.

## **Supplementary Text S5. Mediation analysis output and Directed Acyclic Graphs (DAG)**

**Hypothesis 1 (DAG 1)**

Investigate Mediator: Private Insurance

Exposure: Age

Outcome: Dual sensory impairment (>25 dB HL)


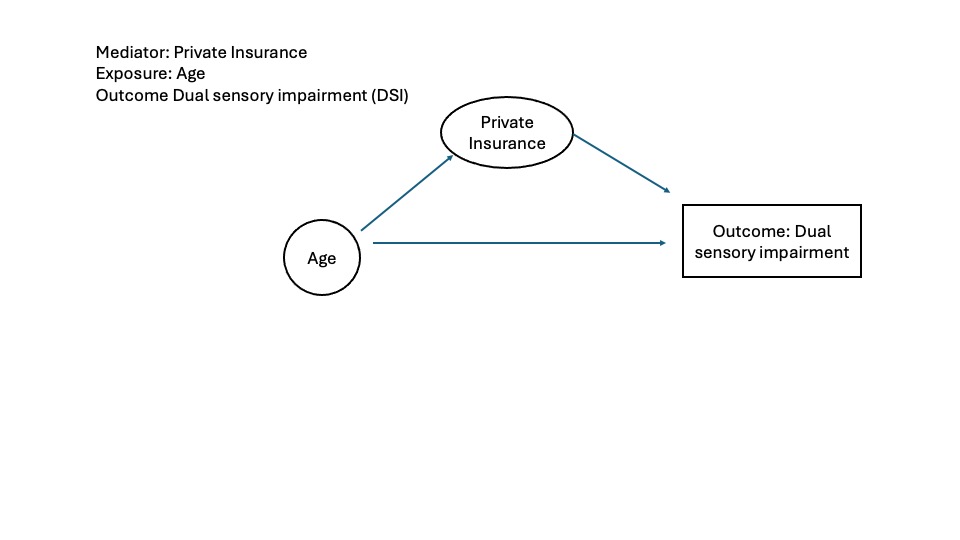


Causal Mediation Analysis

Quasi-Bayesian Confidence Intervals

Output Based on Overall Averages Across Groups

| **Effect** | **Estimate** | **95% CI Lower** | **95% CI Upper** | **p-value** |
| --- | --- | --- | --- | --- |
| ACME (control) | -1.3 × 10⁻⁷ | -1.4 × 10⁻⁶ | 8.2 × 10⁻⁷ | 0.716 |
| ACME (treated) | -1.4 × 10⁻⁷ | -1.5 × 10⁻⁶ | 9.0 × 10⁻⁷ | 0.716 |
| ADE (control) | 4.7 × 10⁻⁶ | 9.0 × 10⁻⁷ | 1.4 × 10⁻⁵ | <0.001 |
| ADE (treated) | 4.7 × 10⁻⁶ | 8.9 × 10⁻⁷ | 1.4 × 10⁻⁵ | <0.001 |
| Total Effect | 4.5 × 10⁻⁶ | 8.5 × 10⁻⁷ | 1.3 × 10⁻⁵ | <0.001 |
| Prop. Mediated (control) | -2.7 × 10⁻² | -2.1 × 10⁻¹ | 1.1 × 10⁻¹ | 0.716 |
| Prop. Mediated (treated) | -3.0 × 10⁻² | -2.3 × 10⁻¹ | 1.2 × 10⁻¹ | 0.716 |
| ACME (average) | -1.3 × 10⁻⁷ | -1.4 × 10⁻⁶ | 8.6 × 10⁻⁷ | 0.716 |
| ADE (average) | 4.7 × 10⁻⁶ | 9.0 × 10⁻⁷ | 1.4 × 10⁻⁵ | <0.001 |
| Prop. Mediated (average) | -2.9 × 10⁻² | -2.2 × 10⁻¹ | 1.2 × 10⁻¹ | 0.716 |

**Abbreviations:**
ACME = Average Causal Mediation Effect; ADE = Average Direct Effect; Prop. Mediated = Proportion mediated.

Sample Size Used: 3573

Simulations: 1000

**Hypothesis 1**

Mediator: Private Insurance

Exposure: Age

Outcome Dual sensory impairment (>40 dB HL)

Causal Mediation Analysis

Quasi-Bayesian Confidence Intervals

Output Based on Overall Averages Across Groups

| **Effect** | **Estimate** | **95% CI Lower** | **95% CI Upper** | **p-value** |
| --- | --- | --- | --- | --- |
| ACME (control) | -0.8 × 10⁻⁷ | -8.4 × 10⁻⁷ | 4.7 × 10⁻⁷ | 0.694 |
| ACME (treated) | -0.9 × 10⁻⁷ | -9.0 × 10⁻⁷ | 5.0 × 10⁻⁷ | 0.694 |
| ADE (control) | 2.5 × 10⁻⁶ | 1.9 × 10⁻⁷ | 9.6 × 10⁻⁶ | <0.001 |
| ADE (treated) | 2.5 × 10⁻⁶ | 1.9 × 10⁻⁷ | 9.6 × 10⁻⁶ | <0.001 |
| Total Effect | 2.4 × 10⁻⁶ | 1.9 × 10⁻⁷ | 9.3 × 10⁻⁶ | <0.001 |
| Prop. Mediated (control) | -2.9 × 10⁻² | -2.4 × 10⁻¹ | 1.1 × 10⁻¹ | 0.694 |
| Prop. Mediated (treated) | -3.2 × 10⁻² | -2.6 × 10⁻¹ | 1.2 × 10⁻¹ | 0.694 |
| ACME (average) | -0.8 × 10⁻⁷ | -8.7 × 10⁻⁷ | 4.8 × 10⁻⁷ | 0.694 |
| ADE (average) | 2.5 × 10⁻⁶ | 1.9 × 10⁻⁷ | 9.6 × 10⁻⁶ | <0.001 |
| Prop. Mediated (average) | -3.1 × 10⁻² | -2.5 × 10⁻¹ | 1.2 × 10⁻¹ | 0.694 |

**Abbreviations:**
ACME = Average Causal Mediation Effect; ADE = Average Direct Effect; Prop. Mediated = Proportion mediated.

Sample Size Used: 3573

Simulations: 1000

**Hypothesis 2 (DAG 2)**

Investigate Mediator: Private Insurance

Exposure: Diabetes

Outcome: Dual sensory impairment (>25 dB HL)


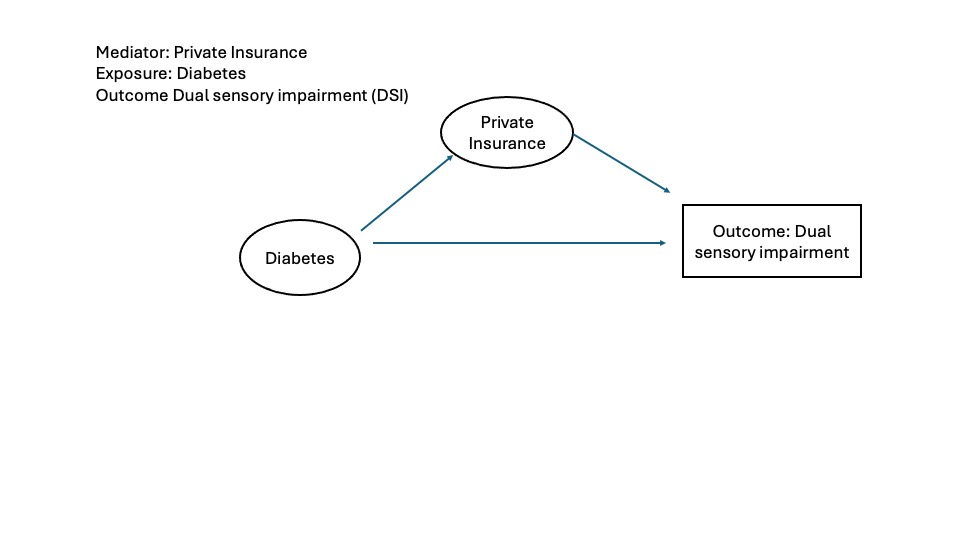


Causal Mediation Analysis

Quasi-Bayesian Confidence Intervals

Output Based on Overall Averages Across Groups

| **Effect** | **Estimate** | **95% CI Lower** | **95% CI Upper** | **p-value** |
| --- | --- | --- | --- | --- |
| ACME (control) | 3.9 × 10⁻³ | 2.0 × 10⁻³ | 6.3 × 10⁻³ | <0.001 |
| ACME (treated) | 6.2 × 10⁻³ | 3.0 × 10⁻³ | 1.0 × 10⁻² | <0.001 |
| ADE (control) | 1.8 × 10⁻² | 2.1 × 10⁻³ | 3.8 × 10⁻² | 0.030 |
| ADE (treated) | 2.1 × 10⁻² | 2.5 × 10⁻³ | 4.3 × 10⁻² | 0.030 |
| Total Effect | 2.5 × 10⁻² | 7.4 × 10⁻³ | 4.7 × 10⁻² | 0.002 |
| Prop. Mediated (control) | 1.6 × 10⁻¹ | 6.1 × 10⁻² | 6.2 × 10⁻¹ | 0.002 |
| Prop. Mediated (treated) | 2.6 × 10⁻¹ | 1.3 × 10⁻¹ | 6.8 × 10⁻¹ | 0.002 |
| ACME (average) | 5.0 × 10⁻³ | 2.6 × 10⁻³ | 8.0 × 10⁻³ | <0.001 |
| ADE (average) | 1.9 × 10⁻² | 2.3 × 10⁻³ | 4.1 × 10⁻² | 0.030 |
| Prop. Mediated (average) | 2.1 × 10⁻¹ | 9.5 × 10⁻² | 6.4 × 10⁻¹ | 0.002 |

**Abbreviations:**

ACME = Average Causal Mediation Effect; ADE = Average Direct Effect; Prop. Mediated = Proportion mediated.

Sample Size Used: 3573

Simulations: 1000

**Hypothesis 2 (DAG 2)**

Investigate Mediator: Private Insurance

Exposure: Diabetes

Outcome Dual sensory impairment (>40 dB HL)

Causal Mediation Analysis

Quasi-Bayesian Confidence Intervals

Output Based on Overall Averages Across Groups

| **Effect** | **Estimate** | **95% CI Lower** | **95% CI Upper** | **p-value** |
| --- | --- | --- | --- | --- |
| ACME (control) | 1.9 × 10⁻³ | 6.9 × 10⁻⁴ | 3.4 × 10⁻³ | <0.001 |
| ACME (treated) | 4.1 × 10⁻³ | 1.5 × 10⁻³ | 7.8 × 10⁻³ | <0.001 |
| ADE (control) | 1.6 × 10⁻² | 4.3 × 10⁻³ | 3.1 × 10⁻² | 0.002 |
| ADE (treated) | 1.8 × 10⁻² | 4.9 × 10⁻³ | 3.6 × 10⁻² | 0.002 |
| Total Effect | 2.0 × 10⁻² | 7.3 × 10⁻³ | 3.8 × 10⁻² | 0.002 |
| Prop. Mediated (control) | 9.1 × 10⁻² | 2.9 × 10⁻² | 3.3 × 10⁻¹ | 0.002 |
| Prop. Mediated (treated) | 2.1 × 10⁻¹ | 8.2 × 10⁻² | 4.3 × 10⁻¹ | 0.002 |
| ACME (average) | 3.0 × 10⁻³ | 1.2 × 10⁻³ | 5.4 × 10⁻³ | <0.001 |
| ADE (average) | 1.7 × 10⁻² | 4.6 × 10⁻³ | 3.3 × 10⁻² | 0.002 |
| Prop. Mediated (average) | 1.5 × 10⁻¹ | 5.7 × 10⁻² | 3.8 × 10⁻¹ | 0.002 |

**Abbreviations:**
ACME = Average Causal Mediation Effect; ADE = Average Direct Effect; Prop. Mediated = Proportion mediated.

Sample Size Used: 3573

Simulations: 1000

The “Table 2 fallacy” arises when coefficients from a single multivariable model are misinterpreted as causal effects for all included variables. Presenting estimates for both the primary exposure and adjustment covariates together can imply that each coefficient represents a valid total effect, even though variables may lie on different causal pathways and cannot meet the assumptions required for causal interpretation within one model.^3^ To avoid this, Directed Acyclic Graphs (DAGs) and mediation analysis was used to evaluate the hypothesised causal pathways and identify mediators contributing to the total effect through indirect effects. When mediators were identified, separate regression models were fitted to estimate the total effect appropriately and prevent misinterpretation. Age, diabetes, and private insurance were specified as the primary exposures for DSI. Causal pathways and potential mediators were defined using DAGs and each mediating relationship was examined using the *mediation* package in R, which implements a model‑based causal mediation framework.^4^

The mediation analysis for the DAG 1 was not statistically significant, indicating that there is no mediation effect. In DAG 2, mediation analysis revealed that the mediation effect is statistically significant (p<0.001) with the estimated mediation effect (indirect) account for 21% and 15% of the total effect for DSI with definition >25 dB HL and >40 dB HL, respectively. DAG 1 mediation analysis indicates that “Table 2 fallacy” is unlikely but not for DAG 2 mediation analysis. We performed analysis of DAG 2 to investigate the “table 2 fallacy” with “diabetes” as our primary exposure of interest and “private insurance” as the intermediate factor, and all other factors were assumed to be confounders for both DSI definition.

We analysed two models for each definition: with and without “private insurance”. The results for definition of >25 dB HL were odds ratio (OR): 1.42 (95% CI: 0.88-2.23) and 1.54 (95% CI 0.96-2.41), respectively. For the definition >40 dB HL, OR 1.86 (95% CI 0.98-3.40) and 2.01 (95% CI 1.07-3.68), respectively. Despite the slight differences in the OR where it indicates the indirect effect indeed contribute to the total effect, it does not change the final interpretation due to a minimal difference in OR and it is not statistically significant. Hence, our original findings can be interpreted as an estimation of the total effect specific to each of the covariates.

**References**

1. Huria T, Palmer SC, Pitama S, et al. Consolidated criteria for strengthening reporting of health research involving Indigenous peoples: the CONSIDER statement. BMC Med Res Methodol. 2019;19(1):173.

2. Harfield S, Pearson O, Morey K, et al. Assessing the quality of health research from an Indigenous perspective: the Aboriginal and Torres Strait Islander quality appraisal tool. BMC Med Res Methodol. 2020;20(1):79.

3. Westreich D, Greenland S. The table 2 fallacy: presenting and interpreting confounder and modifier coefficients. Am J Epidemiol. 2013;177(4):292-8.

4. Tingley D, Yamamoto T, Hirose K, Keele L, Imai K. mediation: R package for causal mediation analysis. J Stat Softw. 2014;59(5):1-38.
